# Is rate-dependent perception affected by linguistic information about the intended syllable rate?

**DOI:** 10.3758/s13423-025-02746-x

**Published:** 2025-09-25

**Authors:** Giulio G. A. Severijnen, Hans Rutger Bosker, James M. McQueen

**Affiliations:** 1 Donders Institute for Brain, Cognition, and Behavior, Radboud University, Thomas van Aquinostraat 4, 6525 GD Nijmegen, The Netherlands; 2 Max Planck Institute for Psycholinguistics, PO Box 310, 6500 AH Nijmegen, The Netherlands

**Keywords:** Rate-dependent perception, Rate normalization, Syllable rate, Linguistic information

## Abstract

Speech is highly variable in rate, challenging the perception of sound contrasts that are dependent on duration. Listeners deal with such variability by perceiving incoming speech relative to the rate in the surrounding context. For instance, the same ambiguous vowel is more likely to be perceived as being long when embedded in a fast sentence, but as short when embedded in a slow sentence. However, it is still debated to what extent domain-general and domain-specific mechanisms (i.e., language- or speech-specific mechanisms) contribute to rate-dependent perception. Here we examined the role of domain-specific mechanisms in an implicit rate-normalization task in which we manipulated linguistic knowledge about how many syllables words have. Dutch participants were presented with lists of Dutch words that were acoustically ambiguous with regard to having one or two syllables (e.g., /k?ˈlɔm/ can be monosyllabic *klom,* /klɔm/, or bisyllabic *kolom,* /ko.ˈlɔm/). While being presented with these ambiguous word lists, they saw monosyllabic or bisyllabic transcriptions of the lists on the screen. We predicted that the same acoustic stimulus would be perceived as faster (more syllables per second) when combined with bisyllabic orthography compared to monosyllabic orthography. In turn, this would lead to downstream influences on vowel length perception in target words embedded within the word lists (rate-dependent perception of Dutch /ɑ/ vs./ /aː/). Despite evidence of successful orthographic disambiguation of the ambiguous word lists, we did not find evidence that linguistic knowledge influenced participants’ rate-dependent perception. Our results are best accounted for by a domain-general account of rate-dependent perception.

**Supplementary Information:**

The online version contains supplementary material available at 10.3758/s13423-025-02746-x.

## Introduction

Speech is highly variable in rate. For example, speech rates can differ depending on the language one speaks (Pellegrino et al., 2011), phrase length, dialectal differences, and differences between individual talkers (Quené, 2008). Such variability is problematic for speech perception since many sound contrasts depend on duration. For example, in the sentence “He found a pear/bear,” the voice onset time (VOT) of the plosive in “pear/bear” will vary depending on the speech rate in the sentence (Allen & Miller, 2001; Miller, 1981). Listeners thus have to take these differences in speech rate into account to correctly perceive the intended sounds. The present study examined to what extent listeners use different types of information about speech rate (acoustic, linguistic) in dealing with differences in speech rate.

Listeners can deal with differences in speech rate by perceiving incoming speech relative to the rate in the surrounding context. This process, called rate-normalization or rate-dependent speech perception (for review, see Stilp, 2020), affects the perception of speech cues such as VOT (King et al., 2024; Miller & Dexter, 1988; Miller & Liberman, 1979; Summerfield, 1981; Toscano & McMurray, 2015), formant transitions (Wade & Holt, 2005), vowel length (Bosker & Reinisch, 2015; Maslowski et al., 2019, 2020), and consonant length (Heffner et al., 2024), but also perception of lexical stress (Reinisch et al., 2011a) and word segmentation (Dilley & Pitt, 2010; Heffner et al., 2013). In all cases, rate-dependent perception is a contrastive process: speech in a fast context is perceived as relatively slow (i.e., having a longer duration) but the same speech in a slow context as relatively fast (i.e., having a shorter duration). Thus, for example, perception of an ambiguous Dutch vowel between short /ɑ/ and long /aː/ will be biased towards /aː/ when presented in a fast context, and vice versa (Bosker, 2017).

An ongoing debate in the literature on rate-dependent perception concerns to what extent domain-general, auditory mechanisms and domain-specific (i.e., language- or speech-specific) mechanisms contribute to rate-dependent perception. Thus far, evidence has been found in support for contributions of both mechanisms. Evidence in favor of domain-general accounts comes from studies showing rate-dependent perception in non-human species (Welch et al., 2009) and, in human listeners, in response to non-speech stimuli (Bosker, 2017; Diehl & Walsh, 1989; Wade & Holt, 2005). Moreover, rate-dependent perception occurs prelexically (Reinisch et al., 2011b; Reinisch & Sjerps, 2013), is an automatic process that is affected by non-target talkers (Newman & Sawusch, 2009), and is not modulated by attentional processes (Bosker et al., 2020) or cognitive load (Bosker et al., 2017).

Evidence in favor of domain-specific accounts comes from studies showing that rate-dependent perception involves speech-specific mechanisms such as listener expectations about the to-be-perceived speech rate based on linguistic information. For example, the speech rate of sentences spoken at the same rate is perceived differently depending on whether they are spoken in a native or a foreign language (Bosker & Reinisch, 2015, 2017), whether they contain segmental deletions, which are typical for fast speech (Reinisch, 2016), or depending on the comprehensibility of the sentence (Chen et al., 2025). Yet others have focused on the lexical rate effect (LRE), through which differences in speech rate affect the perception of the presence of words and word boundaries (Baese-Berk et al., 2019; Dilley & Pitt, 2010). It has been shown that this effect interacts with linguistic knowledge about the grammaticality of utterances (Morrill et al., 2015) and is specific to intelligible precursors and not elicited by degraded speech precursors (Pitt et al., 2016). In sum, there is evidence for rate-dependent perception to be driven by a low-level, auditory component and additionally by a higher-level component that can be guided by linguistic information.

In an attempt to gauge the relative contribution of domain-general and domain-specific mechanisms, previous research examined how syllable rate (i.e., the number of syllables per second) affects rate-dependent perception while reducing effects from the acoustic input (Severijnen et al., 2023). Dutch participants were presented with word lists that had the same duration (removing duration as a possible cue to speech rate), but differed in whether they contained monosyllabic or bisyllabic non-target (i.e., context) words (e.g., monosyllabic: “/ɤlet, trɛin, brɛit, **stɑt/staːt**, klɔm/” vs. bisyllabic: “/ɤə.ˈlet, tɛ.ˈrɛin, bə.ˈrɛit, **stɑt/staːt**, ko.ˈlɔm/”; the target word is depicted in bold). Vowel perception in the target word (e.g., stɑt/staːt) was taken as an implicit measure of rate-dependent perception. Results showed that bisyllabic word lists (with more syllables per unit time, and hence faster) induced more long vowel responses compared to the monosyllabic word lists of the same duration (with half the number of syllables per unit time), suggesting that even when the duration of the context is identical, the syllable rate affected rate-dependent perception. However, while this manipulation eliminated overall word duration as a possible acoustic cue to speech rate, rate-dependent perception was presumably still driven by pronounced acoustic differences in syllable durations between the two (word duration-matched) monosyllabic versus bisyllabic conditions.

Despite this dominance of acoustics in rate-dependent perception, experiments using explicit rate judgements have shown that listeners do rely on non-acoustic, linguistic knowledge about the *intended* (instead of *acoustic*) syllable rate when evaluating speech rate (Chen et al., 2025; Koreman, 2006; Plug et al., 2022, 2023). For example, Plug et al. (2023) presented English participants with sentences containing words that were ambiguous with regard to being monosyllabic and bisyllabic (e.g., between “sport” vs. “support”), by replacing the first vowel in the bisyllabic word (“support”) with a pre-stress schwa. Auditory presentation of these sentences was accompanied by orthographic transcriptions of the monosyllabic word form (e.g., “sport”) or the bisyllabic word form (e.g., “support”). Trials with the bisyllabic word form were rated as faster than trials with the monosyllabic word form, despite involving the identical auditory stimulus, suggesting that participants are guided by the intended phone rate, even when controlling for differences in the acoustic rate. However, the authors also reported that this effect was quite fragile and only found in a subset of the trials. Specifically, only when participants were presented with trials in which the overall sentence duration was not an informative cue to speech rate, or when the target sentence was presented last (i.e., after a reference sentence and not before it), participants used the orthographic transcriptions to guide their judgements. This suggests that when the linguistic effect arises, it is a subtle effect which might be easily affected by other cues to speech rate, cognitive load, or short-term memory constraints.

Given these effects of orthographic information on speech rate perception in explicit tasks and previous other findings showing that rate-dependent perception can partly be driven by domain-specific information (Bosker & Reinisch, 2015, 2017; Chen et al., 2025; Morrill et al., 2015; Pitt et al., 2016; Reinisch, 2016), the present study examined whether linguistic information cued through orthography affects rate-dependent perception in an implicit task. More specifically, we ask: Does the number of intended syllables (i.e., syllable rate), cued by orthographic transcriptions, affect rate-dependent perception in acoustically identical word lists?

It is important to note that explicit rate judgements (as used in Plug et al., 2023) differ from implicit tasks that test rate-dependent perception in mainly two ways. First, in explicit rate judgements, participants’ attention is explicitly guided towards the speech rate in the input, while implicit tasks measure rate perception indirectly (e.g., through perception of a target word in the input). Second, the responses given in explicit rate judgements can be considered to be the result of post-perceptual decisions, where a comparison is made between the expected and perceived sound (Bosker et al., 2017), while implicit tasks could reflect prelexical processing alone (Reinisch & Sjerps, 2013). For these reasons, using explicit rate judgements might lead listeners to use cues that are otherwise ignored during on-line speech perception. Thus, it remains uncertain whether results from explicit rate judgements translate to implicit tasks that more strongly reflect prelexical processing. Indeed, there is mixed evidence regarding whether explicit and implicit tasks show the same pattern of results (Chen et al., 2025; Reinisch, 2016; Steffman & Jun, 2021).

Another reason to question whether effects of orthography would translate to rate-dependent perception in an implicit task is that it is unclear whether orthography affects speech perception on-line (Ziegler et al., 2003) or at a post-perceptual decision stage (Cutler et al., 1998; Pattamadilok et al., 2007). Interestingly, however, experiments using event-related potentials (ERPs), which can be taken as an informative measure for on-line processing, have shown that inconsistencies between orthographic and auditory input of words affected speech perception on-line, and even before any effects of word frequency, which is a classic marker of lexical access (Perre et al., 2009; Perre & Ziegler, 2008). Therefore, it seems possible that orthography could affect speech perception on-line and thus affect rate-dependent perception at the prelexical stage.

In this study, we asked if orthographic information can modulate rate-dependent perception in an implicit task. We built on the results in Plug et al. (2023), and implemented this in Experiment 2 following the design in Severijnen et al. (2023). More specifically, we took the same word lists as in Severijnen et al. (2023) (e.g., “/ɤlet, trɛin, brɛit, **stɑt/staːt**, klɔm/” vs. “/ɤə.ˈlet, tɛ.ˈrɛin, bə.ˈrɛit, **stɑt/staːt**, ko.ˈlɔm/”) and created an ambiguous condition, midway between being monosyllabic and bisyllabic, by compressing the duration of the first vowel in bisyllabic words to be ambiguous between schwa absent versus present (e.g., “/ɤ?ˈlet, t?ˈrɛin, b?ˈrɛit, **stɑt/staːt**, k?ˈlɔm/.” Similar to the procedure in Plug et al. (2023), we then presented participants with orthographic transcriptions indicating whether the list should be perceived as mono- or bisyllabic. Critically, in Experiment 2, the word lists contained tokens of target word vowel continua (e.g., stɑt/staːt), ranging from a short /ɑ/ to a long /aː/, and participants had to indicate which target word they had heard on each trial. We predicted that acoustically ambiguous word lists paired with bisyllabic orthography (henceforth the “ambiguous-as-bisyllabic” condition) would be perceived as faster than when paired with monosyllabic orthography (the “ambiguous-as-monosyllabic” condition), leading to a higher proportion of long vowel responses (rate-dependent perception on Dutch /ɑ/ vs. /aː/). However, before we ran Experiment 2, we first assessed the suitability of our stimuli in Experiment 1.

## Experiment 1: Word recognition

### Method

#### Rationale

We first tested whether presenting orthographic transcriptions of monosyllabic and bisyllabic word lists had the intended effect of disambiguating acoustically ambiguous word lists. Specifically, participants heard ambiguous word lists that were paired with the orthographic transcription of a monosyllabic or a bisyllabic word list, and responded as to whether they heard a monosyllabic or bisyllabic context word (e.g., “did you hear *klom* or *kolom?*”).

We hypothesized that participants would more often indicate hearing a bisyllabic context word (e.g., *kolom*) when they heard ambiguous word lists and saw bisyllabic orthography compared to when they heard ambiguous word lists and saw monosyllabic orthography.

#### Participants

We recruited 20 native speakers of Dutch (14 female, six male; *M*_*age*_ = 25.25, range = 18–35 years) from the Prolific participant pool (Palan & Schitter, 2018). All participants reported that they did not have any hearing difficulties and gave informed consent as approved by the Ethics Committee of the Faculty of Social Sciences of Radboud University (project code: ECSW-LT-2022-4-14-27223), and were paid for their participation.

##### Stimuli

We designed the stimuli of Experiment 1 with Experiment 2 in mind, meaning that stimuli were shared across experiments. Consequently, stimuli needed to fit the requirements of both Experiment 1 (ambiguous context words) and Experiment 2 (target words from an /ɑ-aː/ continuum). Hence, the stimuli consisted of word lists containing four context words and one target word (*note:* this is the target word for Experiment 2) that appeared in pre-final position (e.g., “*gleed, trein, breit, ****stad/staat****, klom*”; target word is depicted in bold). The word lists appeared in three conditions: a monosyllabic condition, corresponding to a slow rate (e.g., “*gleed, trein, breit****, stad/staat****, klom*”*;* “/ɤlet, trɛin, brɛit, **stɑt/staːt**, klɔm/”; “slid, train, knits, city/state, climbed”), a bisyllabic condition, corresponding to a fast rate (e.g., “*geleed, terrein, bereid, ****stad/staat****, kolom*”*;* “/ɤə.ˈlet, tɛ.ˈrɛin, bə.ˈrɛit, **stɑt/staːt**, ko.ˈlɔm/”; “articulated, terrain, willing, city/state, column”), and an ambiguous condition with the duration of the first vowel in the bisyllabic word (e.g., /o/ in ko.ˈlɔm/) compressed to be ambiguous between schwa present versus absent, corresponding to a rate midway between slow and fast (“*g?leed, t?rein, b?reid, ****stad/staat****, k?lom*”*,* “/ɤ?ˈlet, t?ˈrɛin, b?ˈrɛit, **stɑt/staːt**, k?ˈlɔm/”). To create these lists, we first recorded the context and target words separately, and these were subsequently manipulated. Then the words were combined to create each list.

##### Context words

For the context words, we selected ten minimally different Dutch word pairs in which one word was monosyllabic and the other word was bisyllabic with final stress (e.g., *klom, */klɔm/vs.* kolom, */ko.ˈlɔm/). These differed only in the insertion of an unstressed vowel in the first syllable of the bisyllabic word (for the complete set of context words, see Online Supplementary Material (OSM) Table S1). We recorded a female native talker of Dutch, who produced the words in isolation.

**Fig. 1:**
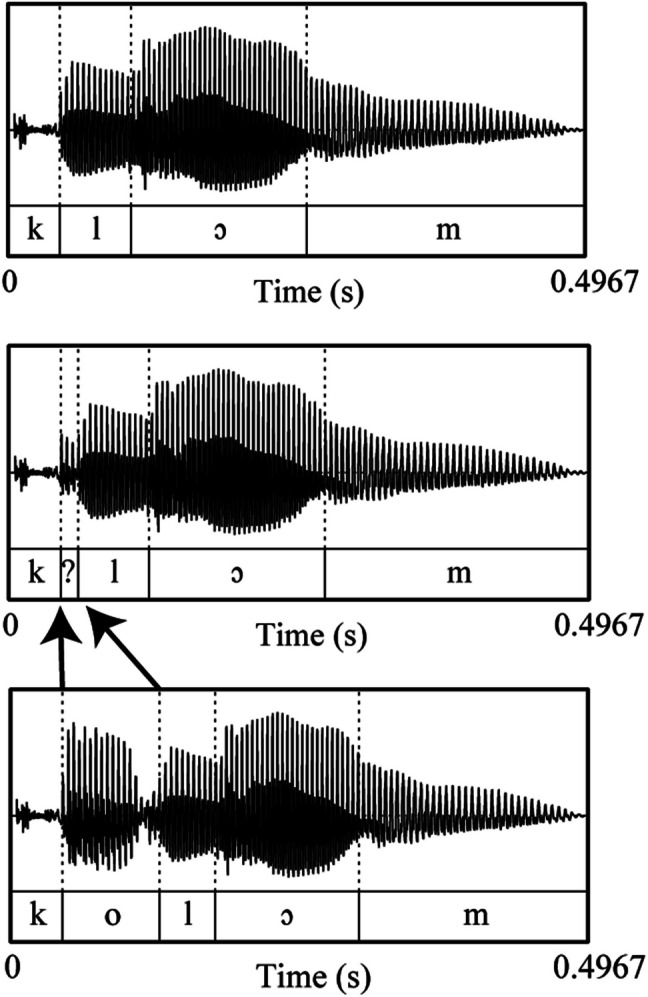
Oscillograms of one of the monosyllabic (**top row**), ambiguous (**middle row**), and bisyllabic (**bottom row**) context words. The vertical lines in each oscillogram show the phoneme boundaries. The arrows between bisyllabic and the ambiguous oscillogram indicate that the first vowel in the bisyllabic word was shortened to create the ambiguous word

Next, we needed three versions of the words (see Fig. 1). Next to the clear monosyllabic and bisyllabic versions, which were slightly adapted versions of the original recordings, we created a third version in which the duration of the unstressed vowel was compressed to be ambiguous between schwa present versus absent, with the intention of making the word ambiguous between mono- and bisyllabic. Additionally, all three versions had to be of the same duration within each word (e.g., *klom, k?lom,* and *kolom* had to be equal in duration) to remove overall word duration as an acoustic cue to speech rate. To create these three versions, we first set the mono- and bisyllabic word of each pair to the same mean duration of the pair using PSOLA in Praat (Boersma & Weenink, 2019). Then we took the bisyllabic version and created an 18-step vowel continuum in which we reduced the duration of the unstressed vowel in 18 equal steps from the vowel duration in the bisyllabic word (step 18) to that in the monosyllabic word (i.e., zero; step 1). With each step, the duration of the second syllable was then increased by the same amount to keep the duration of the entire word constant. Note that the unstressed vowel in these tokens did not have the spectral makeup of a real schwa, but that of an unstressed vowel. Instead, we aimed to approximate a schwa by temporally compressing the vowel.

We then piloted eight steps of the 18-step continua in a two-alternative forced-choice (2AFC) pilot with 24 participants who did not participate in either experiment reported here (Pilot 1). We tested steps 1 and 18 (i.e., clear mono- and bisyllabic versions), and the six most ambiguous steps, based on auditory evaluations of the first author (steps 3–8). Based on the results of this pilot, we selected the most ambiguous step, unique for each context word. This was confirmed by calculating the mean proportion of bisyllabic word responses given on the selected steps (*M* = 0.52, *SD* = 0.017). For details on Pilot 1, see OSM section 1.1.

##### Target words

Target words were included in the present design to meet the requirements for Experiment 2; they were unimportant for Experiment 1. The target words were six Dutch monosyllabic minimal pairs differing only in whether the word contained a short or a long vowel (e.g., *stad*, /st**ɑ**t/vs. *staat*, /staːt/). For the complete set of target words, see OSM Table S2. The words were recorded in isolation by the same female native speaker of Dutch.

We then created a duration continuum for each word pair, ranging from a short /ɑ/ (step 1) to a long /aː/ (step 7). Since in Dutch, the /ɑ-aː/ vowel contrast is cued by both spectral and durational cues (Adank et al., 2004), we selected ambiguous values for the first and second formant (F1; F2). Using Burg’s LPC method in Praat, we set the F1 to 777 Hz and the F2 to a unique ambiguous F2 value for each target word (ranging from 1,354 Hz to 1,501 Hz, see OSM Table S5). These values were shown to be perceptually close to ambiguous based on a second pilot (for details on the target word manipulations and Pilot 2, see OSM sections 1.2–1.3). For the duration continuum, we first measured the average vowel duration in a short /ɑ/ (115 ms) and a long /aː/ (247 ms). We then manipulated vowel length using PSOLA in Praat, and created, based on auditory evaluations from the first author, a duration continuum which ranged from 72 ms for a short /ɑ/ (step 1) to 203 ms for a long /aː/ (step 7). Finally, to increase the unambiguity of the extreme steps (steps 1 and 7), these were set to F2 values that indicated a clear /ɑ/ or /aː/, again with unique values for each target word (see OSM Table S5). Based on the results from Pilot 2, we then selected the two unambiguous steps (steps 1 and 7) and three middle, ambiguous steps (steps 3, 4, and 5) for the experiment.

##### Word lists

The above manipulations resulted in ten context word triplets, containing one clear monosyllabic, one clear bisyllabic, and an ambiguous version, all equal in duration. Further, we had six target word duration continua, each consisting of two unambiguous steps (one clear short vowel, one clear long vowel) and three middle steps that were ambiguous between a short and a long vowel.

Next, we created the word lists. Each list contained four context words, and one target word in pre-final position. We created six different combinations of context words (see OSM Table S3), by sampling four different context words from the complete set of context words. Critically, for every monosyllabic version, there was a bisyllabic and an ambiguous version (e.g., monosyllabic: *gleed, trein, breit, ****stad/staat****, klom*”*;* bisyllabic: “*geleed, terrein, bereid, ****stad/staat****, kolom*”*;* ambiguous: “*g?leed, t?rein, b?reid, ****stad/staad****, k?lom*”). Each word list combination was then linked to one target word pair, and we spliced each step of the five-step continuum for that word in the pre-final position of the list. Each list thus contained five words, which were concatenated into lists with 50 ms of silence between the words. Recall that each context word had the same duration in the three conditions (monosyllabic, bisyllabic, and ambiguous). Hence, the word lists were also equally long across the three conditions. The final stimulus set consisted of a total of 90 unique stimuli (6 word lists $\times$ 3 conditions $\times$ 5 duration steps).

##### Procedure

The experiment was built and hosted on the Gorilla Experiment Builder (www.gorilla.sc). First, participants performed a headphone screening test (Woods et al., 2017), in which three pure tones were dichotically presented and participants were instructed to select the quietest one. One of the pure tones was presented 180° out of phase across the two stereo channels, which makes the task easy when wearing headphones but difficult over speakers due to phase cancellation. The task aimed to ensure that the majority of participants were wearing headphones during the experiment, as shown by Woods et al. (2017), who found 65% accuracy in detecting headphone versus speaker users. Only participants who passed the headphone screening (at least five out of six correct trials; 20 out of 35 participants who started the experiment) could continue with the experiment proper.

In the experiment, participants were auditorily presented with the word lists and visually presented with the orthographic transcription of those lists. They were instructed to indicate for one of the *context* words which word they had heard (e.g., *klom* or *kolom*). They did not know in advance of the trial which context word they had to respond to; the two alternatives were presented for a 2AFC decision at offset of the auditory list. On each trial, participants therefore responded to one of the context words in the list, but we collected responses for all four context words on separate trials. Since we focused on perception of the context words (i.e., the target words were irrelevant to the task at hand), we did not need responses on all steps of the five-step target word continua. Thus, we only presented four out of the five steps, which was rotated across target words, ensuring that there was a relatively uniform distribution of which steps were presented in the experiment (note that due to a scripting error there were more repetitions of steps 1 and 2 compared to the rest). This resulted in 72 *unique* stimuli presented in this task (3 conditions $\times$ 4 repetitions $\times$ 6 word lists).

Each trial started with a fixation cross in the middle of the screen. After 500 ms, the word list was auditorily presented and at the same time, the orthographic transcription of the word list was visually presented in the middle of the screen. In the orthographic transcription, the target word was replaced with three dots (e.g., “*gleed, trein, breid … klom*”*)*. Crucially, in the ambiguous word list trials, participants always heard the ambiguous word list but either saw the monosyllabic transcription (e.g., “*gleed, trein, breid, … klom*”*)* or the bisyllabic transcription (“*geleed, terrein, bereid, … kolom*”), depending on the block (see below). At sound offset, the two members of one of the context words appeared in the middle of the screen, one left and one right (response position was counterbalanced across participants). Participants were instructed to select one of the options using button presses (Z or M for the left and right option, respectively) at sound offset. If no response was given after 3 s from sound offset, the trial was recorded as a missing data point. The next trial started 1 s after the response or after the timeout in case of a missing data point.

**Fig. 2:**
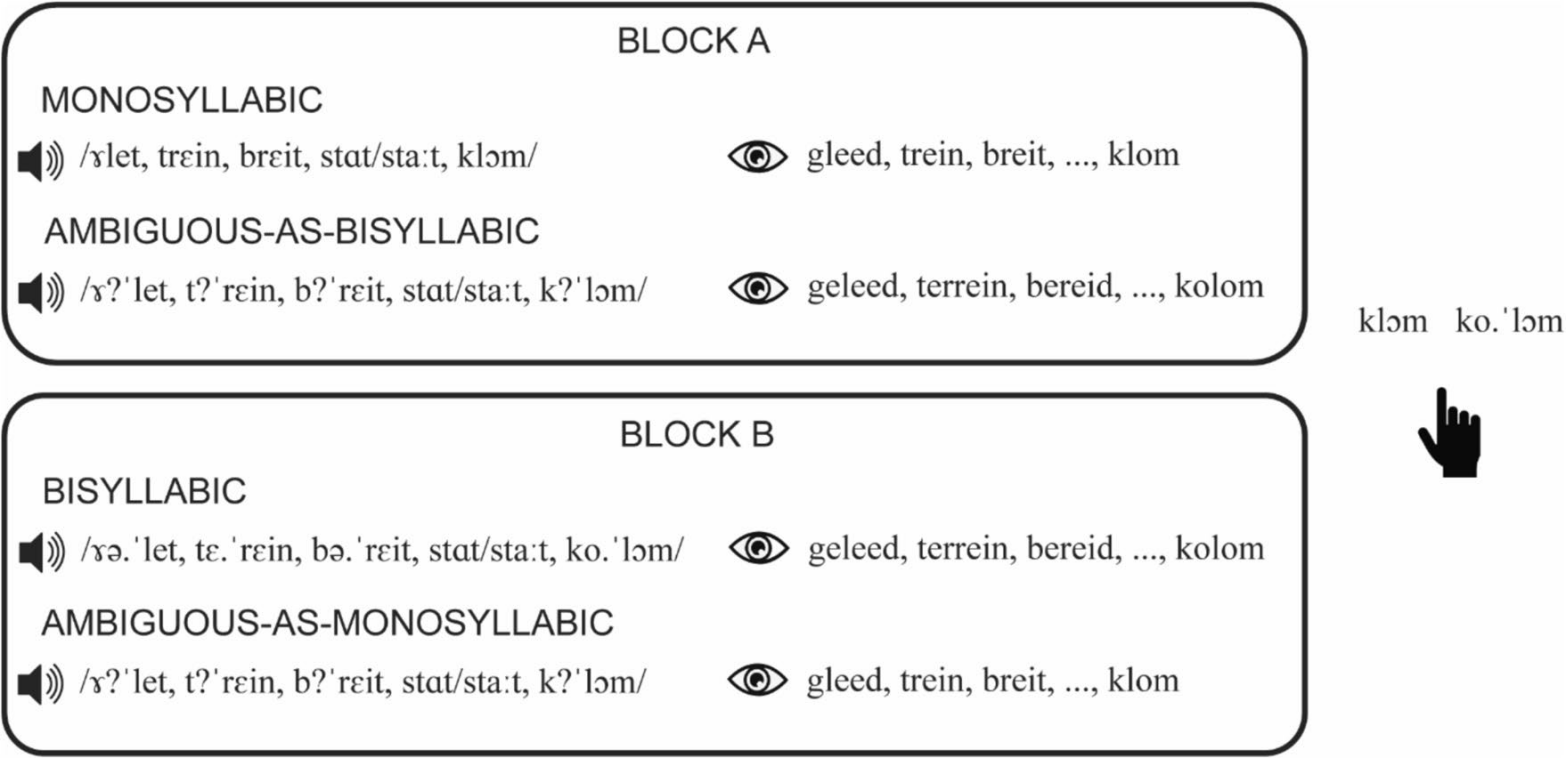
Schematic overview of the word recognition task of Experiment 1. In Block A, participants received monosyllabic trials with clear monosyllabic audio and orthography (e.g., “*gleed, trein, breit, …, klom*”) and ambiguous-as-bisyllabic trials with ambiguous audio and bisyllabic orthography (e.g., “*geleed, terrein, bereid, …, kolom*”). In Block B, participants received bisyllabic trials with bisyllabic audio and orthography and ambiguous-as-monosyllabic trials with ambiguous audio and monosyllabic orthography. On each trial, participants responded with button presses for which context word they had heard. The order of blocks was counterbalanced across participants

The stimuli were presented in two different blocks (see Fig. 2). In Block A, participants were presented with clear monosyllabic trials (monosyllabic audio and orthography) on half of the trials. The other half were ambiguous-as-bisyllabic trials (ambiguous audio, bisyllabic orthography). In Block B, participants were presented with clear bisyllabic trials (bisyllabic audio and orthography) and ambiguous-as-monosyllabic trials (ambiguous audio, monosyllabic orthography). The order of the blocks was counterbalanced across participants. This blocking paradigm was used to enhance the auditory contrast between the clear monosyllabic/bisyllabic and ambiguous trials to further support the perceptual disambiguation of the ambiguous trials (i.e., on top of the orthographic transcriptions). In each block, participants were presented with 96 experimental trials (6 word lists $\times$ 2 conditions $\times$ 4 context words $\times$ 2 repetitions), which were presented in pseudo-randomized order within blocks. The experimental trials were preceded by six practice trials with stimuli that were not used in the experimental trials, and were excluded from statistical analyses. The word lists presented in the practice trials were consistent with the conditions in the first block for each participant. For example, if the participant heard clear monosyllabic and ambiguous-as-bisyllabic stimuli in the first block, the same conditions would be presented in the preceding practice trials.

Participants were instructed to select one of the *context* words they had heard on each trial while looking at the orthography but crucially focusing on the audio. To encourage this and to avoid participants simply selecting the response option purely based on what they remembered about the orthography, we additionally added “mismatch” trials (12 in total, 5.5%). These were trials that contained clear audio but mismatching orthography. Specifically, Block A contained mismatch trials with clear monosyllabic audio but bisyllabic orthography (and vice versa in Block B).

Finally, 12 additional trials (5.5%) were catch trials, which motivated participants to keep looking at the orthographic transcription during the experiment (i.e., not close their eyes). During these trials, participants heard a clear monosyllabic or bisyllabic word list but instead of the orthographic transcription, they saw *DRUK NU OP DE SPATIEBALK* (“PRESS THE SPACE BAR NOW”) on the screen. Participants were instructed to press the space bar (instead of Z or M) at sound offset, which was indicated by a font color change at sound offset. Catch trials occurred on average every seventh trial, with a jitter of maximally two trials to prevent the trials from occurring at a predictable interval. The experiment thus consisted of 216 trials in total (192 experimental trials, 12 mismatch trials, and 12 catch trials).

##### Statistical analyses

Trials with missing data (*n* = 18, 0.4%) were excluded prior to data analysis. Data from two participants were excluded because the responses on the mismatch trials showed that they did not follow the instructions. One participant gave a mean proportion of bisyllabic word responses of 0.80 on trials with monosyllabic audio and bisyllabic orthography and of 0.50 on trials with bisyllabic audio and monosyllabic orthography, and therefore likely based their responses primarily on the orthography. A second participant gave a mean proportion of bisyllabic word responses of 0.33 in both conditions, which is neither in line with the expected direction of the effect when focusing solely on orthography nor with that of focusing solely on the audio. Regarding the catch trials, only one participant responded incorrectly (i.e., pressed Z or M instead of the space bar) on one out of 12 trials (0.46% of total number of catch trials), but we did not exclude this participant from the analyses. The analyses were conducted on the data from the remaining 18 participants, without the mismatch and catch trials, totaling 3,442 observations. We analyzed the data using a generalized linear mixed model (GLMM) with a logistic linking function in the lmerTest package (Kuznetsova et al., 2017) in R (R Core Team, 2020). The model took the categorization of the context words as the binomial dependent variable (bisyllabic word coded as 1; monosyllabic word coded as 0) and contained Condition as fixed factor (categorical predictor with four levels, dummy coded; intercept was the ambiguous-as-monosyllabic condition). The model further included random intercepts for participants and items. The random structure was optimized following the procedure in Bates et al. (2015), which uses Principal Component Analyses (PCA) to obtain the structure that contained the minimally required factors to explain the largest variance.

## Results

**Fig. 3:**
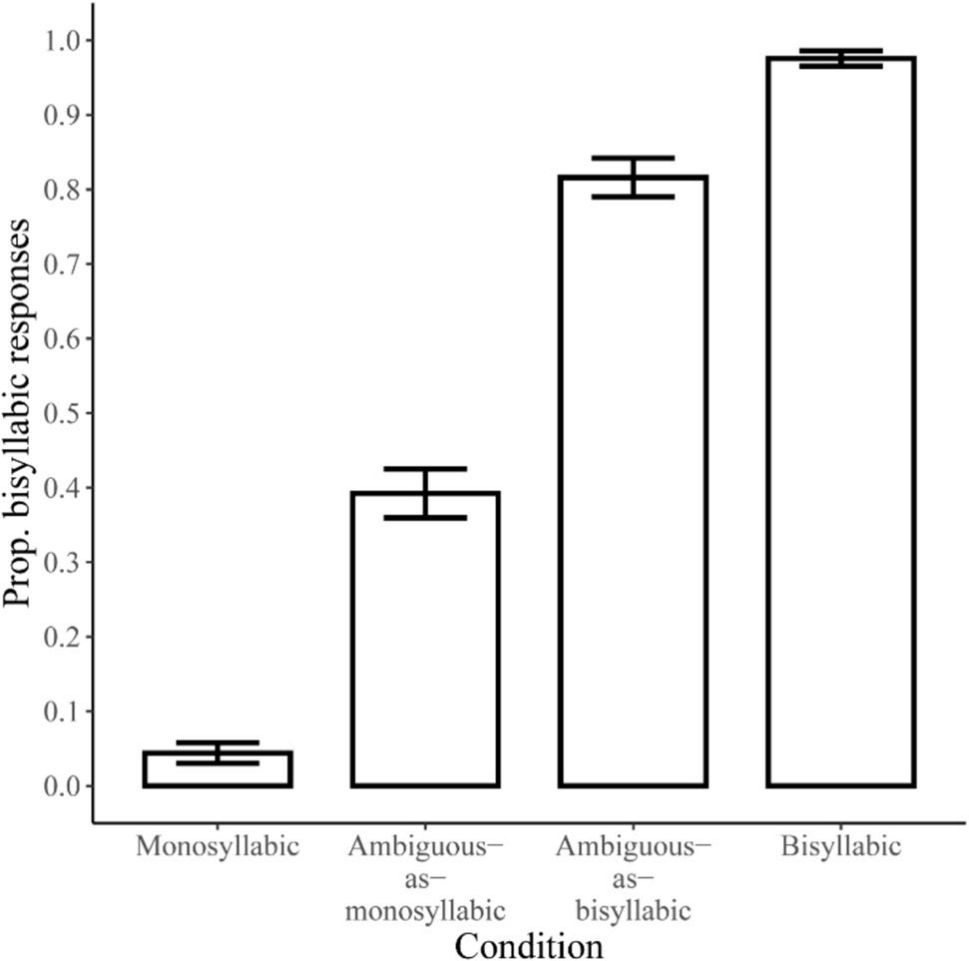
Mean proportions of bisyllabic word responses in the word recognition task of Experiment 1. The results are presented separately for monosyllabic trials (monosyllabic audio, monosyllabic orthography), ambiguous-as-monosyllabic trials (ambiguous audio, monosyllabic orthography), ambiguous-as-bisyllabic trials (ambiguous audio, bisyllabic orthography), and bisyllabic trials (bisyllabic audio, bisyllabic orthography). Error bars represent 95% confidence intervals. As the figure shows, ambiguous word lists with bisyllabic orthography received more bisyllabic responses compared to monosyllabic orthography, confirming that the orthography frequently successfully disambiguated the ambiguous audio

We first examined the proportion of bisyllabic word responses on mismatch trials. On trials with monosyllabic audio and bisyllabic orthography, this proportion was 0.06, while on mismatch trials with bisyllabic audio and monosyllabic orthography it was 0.96, suggesting that participants did indeed follow the instructions and based their responses on the audio. The results for the other conditions are given in Fig. 3. The figure suggests that on ambiguous-as-bisyllabic trials, participants gave more bisyllabic responses compared to ambiguous-as-monosyllabic trials.

The GLMM output (for complete model output, see OSM Table S6) confirmed that there was a significant difference between the ambiguous-as-monosyllabic condition and the ambiguous-as-bisyllabic condition (*β* = 2.626, *SE* = 0.148, *z =* 17.765, *p < *.001). Participants were thus more likely to perceive a bisyllabic context word when they were presented with ambiguous word lists and bisyllabic orthography compared to the same ambiguous audio and monosyllabic orthography.

### Experiment 2: Rate-dependent perception

#### Method

##### Rationale

Experiment 1 confirmed that orthographic disambiguation of ambiguous word lists successfully guided word recognition. This orthographic effect may, in turn, possibly affect the perceived syllable rate. Therefore, Experiment 2 applied the same disambiguation to test whether the perceived syllable rate, cued through orthography, in acoustically identical word lists contributes to rate-dependent perception effects in an implicit task: that is, changing vowel perception in Dutch words containing a short /ɑ/ versus a long /aː/ (e.g., *stad* vs. *staat*, “city” vs. “state”). Specifically, participants heard ambiguous-as-monosyllabic or ambiguous-as-bisyllabic word lists, and indicated whether they heard the target word with a long or a short vowel (e.g., “did you hear *stad* or *staat*?”). We hypothesized that the ambiguous-as-bisyllabic word lists would be perceived as faster and induce a higher proportion of long /aː/ responses compared to the ambiguous-as-monosyllabic word lists.

##### Participants

We recruited 72 native speakers of Dutch (49 female, 23 male; *M*_*age*_ = 23.14, range = 18–35 years). We recruited 32 participants (six female, 23 male; *M*_*age*_ = 21.09, range = 18–30 years) from the Radboud University participant pool and 40 participants (23 female, 17 male; *M*_*age*_ = 24.78, range = 18–35 years) from the Prolific participant pool (Palan & Schitter, 2018). Note that irrespective of the recruitment platform, all participants were presented with the exact same experimental procedure (e.g., hosted online on the Gorilla Experiment Builder). Out of 109 participants who started the experiment, 23 did not pass the headphone screening test (Woods et al., 2017) and an additional 14 dropped out of the experiment during the task. All participants reported that they did not have any hearing difficulties, gave informed consent as approved by the Ethics Committee of the Faculty of Social Sciences of Radboud University (project code: ECSW-LT-2022-4-14-27223), and were paid or received course credits for their participation. None of them had participated in Experiment 1. The sample size was derived from a power analysis (Kumle et al., 2021) which estimated a power of. 840 with 20 participants (see script power_analysis.R on the open data repository). This power analysis was based on the effect size of Pilot 3 that tested the clear monosyllabic versus bisyllabic word lists (see OSM section 1.4 for details on Pilot 3). However, we reasoned that the effect size of the main effect of monosyllabic versus bisyllabic lists would likely be larger than the hypothesized effect of ambiguous-as-monosyllabic versus ambiguous-as-bisyllabic lists. Therefore, we opted for a considerably larger number of participants.

##### Materials and procedure

The procedure was similar to that in Experiment 1. Crucially, it differed in participants not responding to which member of the *context* words they heard in the list, but to which member of the *target* words they heard. This led to the following changes in the stimuli, the trial structure and experiment design.

For the stimuli, we used the same word lists as in Experiment 1 (i.e., the same combination of context words and target words), which appeared in the same conditions as in Experiment 1: monosyllabic (monosyllabic audio and orthography), ambiguous-as-monosyllabic (ambiguous audio, monosyllabic orthography), ambiguous-as-bisyllabic (ambiguous audio, bisyllabic orthography), and bisyllabic (bisyllabic audio, bisyllabic orthography). However, since we now focused on perception of the target words instead of the context words, we needed responses on all steps of the five-step target word continua. This resulted in 90 *unique* acoustic stimuli (6 word lists $\times$ 3 acoustic conditions $\times$ 5 duration steps). Throughout the entire experiment, for each unique target word item (e.g., the *stad-staat* pair), we presented the extreme steps (steps 1 and 7) twice and the middle steps (steps 3, 4, and 5) three times. This was done to increase the number of observations on the critical middle steps while still providing solid anchors of the duration continuum throughout the experiment.

The trial structure was similar to the one in Experiment 1, with one change. At sound offset, two members of one of the *target* words (e.g., *stad* or *staat*) appeared in the middle of the screen instead of the *context* words, one left and one right (position counterbalanced across participants). Participants again were instructed to select one of the options using button presses (Z or M for the left and right option, respectively) at sound offset.

**Fig. 4:**
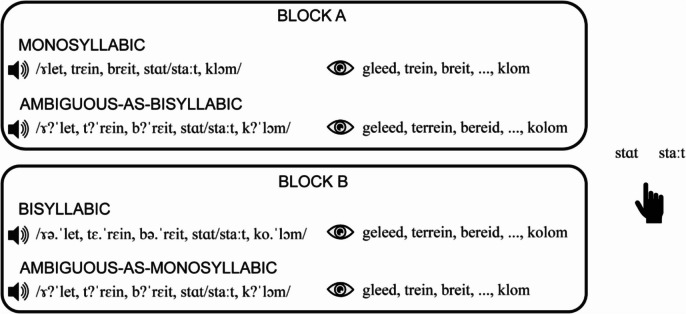
Schematic overview of the implicit rate-dependent perception task of Experiment 2. In Block A, participants received monosyllabic trials with clear monosyllabic audio and orthography (e.g., “*gleed, trein, breit, …, klom*”*)* and ambiguous-as-bisyllabic trials with ambiguous audio and bisyllabic orthography (e.g., “*geleed, terrein, bereid, …, kolom*”*).* In Block B, participants received bisyllabic trials with bisyllabic audio and orthography and ambiguous-as-monosyllabic trials with ambiguous audio and monosyllabic orthography. At each trial, participants responded with button presses for which target word they had heard. The order of blocks was counterbalanced across participants

We again presented the stimuli in two different blocks, similar to Experiment 1 (see Fig. 4), but now we did not include mismatch trials. The reason for this is that the orthographic transcriptions in Experiment 2 did not contain information about which target word was in a trial (the target word was substituted with three dots). Thus, in contrast to Experiment 1, in which participants could solely base their responses on the orthography, this was not possible in Experiment 2. We did still present catch trials (*n* = 48, 13.3%) and the experiment was preceded by six practice trials that had the same conditions as in the first block. In total, the experiment consisted of 312 experimental trials (2 repetitions of two extreme steps $,$ 3 repetitions of three middle steps, 6 target words, 2 conditions, 2 blocks) and 48 catch trials.

##### Statistical analyses

Trials with missing data (*n* = 75, 0.3%) were excluded prior to data analysis. Overall, 48 catch trials received an incorrect response (1.26% of the total number of catch trials), and no participants gave more than three incorrect responses out of 48 catch trials. We did not exclude any participants based on these results. We analyzed the data using a GLMM with a logistic linking function in the lmerTest package (Kuznetsova et al., 2017) in R (R Core Team, 2020). The analyses were conducted on only the three middle duration steps (steps 3, 4, and 5), which equaled 15,494 observations. The model took the categorization of the target words as the binomial dependent variable (long vowel /aː/ coded as 1; short vowel /ɑ/ coded as 0) and contained the following fixed factors: Condition (categorical predictor with four levels, dummy coded; intercept is ambiguous-as-monosyllabic condition), Step (continuous predictor, scaled to z-scores), and Trial Number (continuous predictor, scaled to z-scores). Models with interactions were not included as these did not improve the fit to the model, as tested with log-likelihood comparisons. As in Experiment 1, the random structure was optimized following the PCA procedure in Bates et al. (2015). The final model included by-participant random slopes for Step and Trial number, and by-item random slopes for Trial Number. We ran two additional analyses to check for the following. First, it is possible that analyzing only the middle part of a continuum leads to overestimation of the effect, as the middle steps are perceptually the most ambiguous. Therefore, we also ran models on the data from the full duration continuum (steps 1, 3, 4, 5, and 7). Second, recall that participant recruitment was divided over two recruitment platforms. For comparison, we analyzed the data of the two participant samples separately. The results of both analyses are given in the OSM (sections 1.5 and 1.6). They both revealed a qualitatively similar pattern of results as in the main analyses.

Previewing the results of the GLMM, we did not find evidence for an effect of Condition between the ambiguous-as-monosyllabic and ambiguous-as-bisyllabic conditions. To test for evidence for the null hypothesis (H_0_), we additionally ran two Bayes factor (BF) analyses following Dienes (2014). For the first BF analysis, we specified the prior for H_0_ using the *β* (0.038) and *SE* (0.068) of the ambiguous-as-monosyllabic versus the ambiguous-as-bisyllabic effect in the GLMM output. For the alternative hypothesis (H_1_), we used the larger *β* (0.583) and *SE* (0.089) of the monosyllabic versus bisyllabic effect. We thus tested if there was evidence for an effect in the ambiguous conditions that was as large as that in the unambiguous conditions. However, we reasoned that this effect size might be an overestimate of the expected effect under H_1_. That is, the monosyllabic versus bisyllabic effect is likely primarily driven by acoustics, while the ambiguous-as-monosyllabic versus ambiguous-as-bisyllabic effect cannot be. Therefore, we ran a second, more conservative BF analysis. We computed a new prior for H_1_ based on the GLMM output in Experiment 1. Specifically, we calculated how much smaller the *β* and *SE* were in the ambiguous-as-monosyllabic versus ambiguous-as-bisyllabic effect (*β* = 2.626, *SE* = 0.148) compared to the monosyllabic versus bisyllabic effect (*β* = 8.180, *SE* = 0.315) in Experiment 1 (factor of 0.32 for *β*; factor of 0.47 for the *SE*) and applied these to the estimates of the monosyllabic versus bisyllabic effect in Experiment 2. Similar to the GLMM, we also ran a BF analysis with the estimates obtained from analyses of the entire duration continuum (see OSM section 1.5).

## Results

**Fig. 5:**
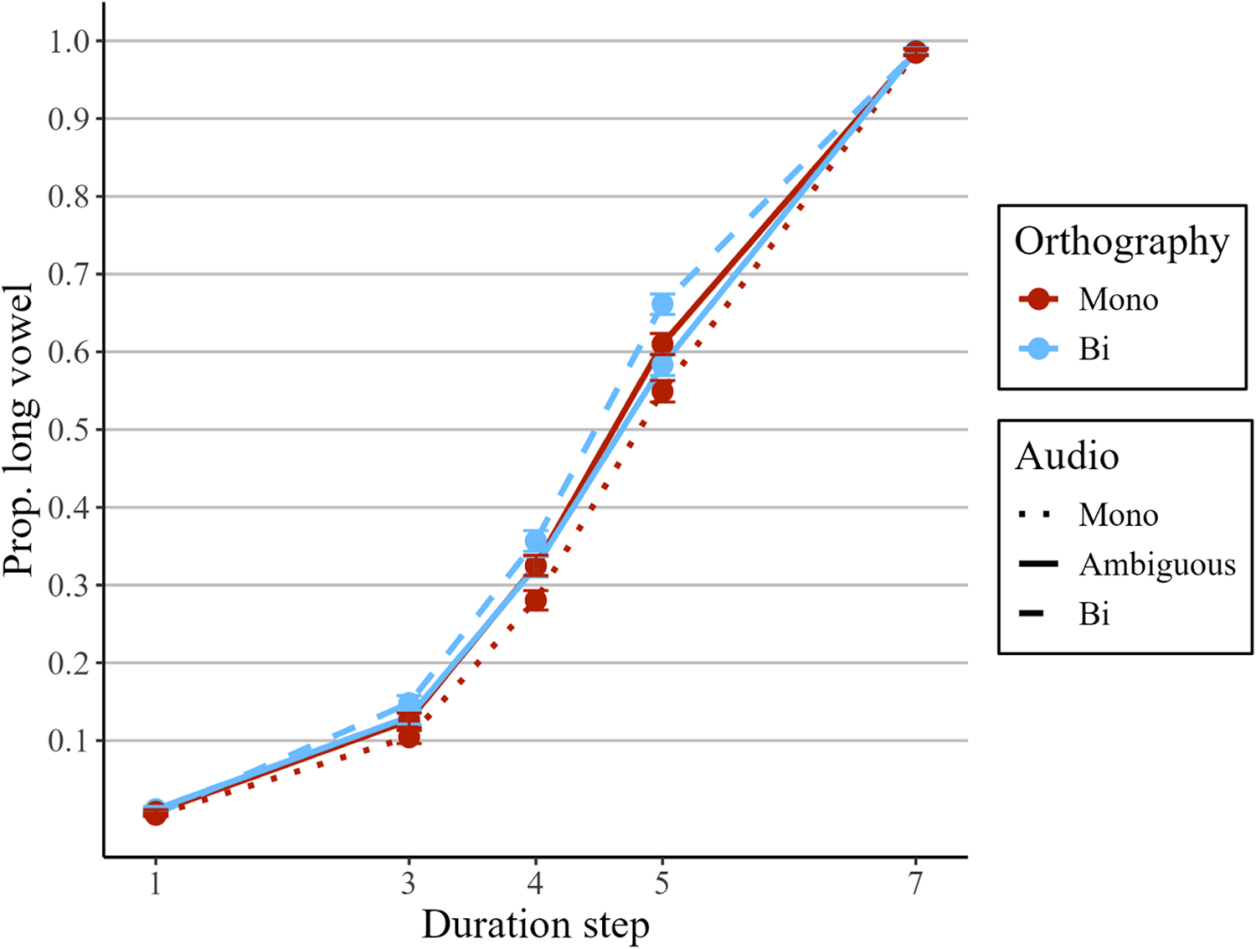
Proportion of long /aː/ responses in the implicit rate-dependent perception task of Experiment 2. The x-axis shows the duration steps from short to long. The blue lines indicate trials with bisyllabic orthography, the red lines with monosyllabic orthography. The dashed line indicates trials with bisyllabic word lists (audio), the dotted line with monosyllabic word lists, and the solid lines ambiguous word lists. Error bars represent 95% confidence intervals. The blue dashed line is higher than the red dotted line, indicating more /aː/ responses in bisyllabic vs. monosyllabic word lists. Crucially, the blue and red solid lines almost completely overlap; that is, there was no evidence of a difference in /aː/ responses between the ambiguous-as-bisyllabic and ambiguous-as-monosyllabic conditions

The proportion of long /aː/ responses on the anchor steps 1 and 7, averaged across conditions, was .007 (step 1) and .98 (step 7), which shows that participants’ performance on these steps was at ceiling/floor and hence that they were indeed effective perceptual anchors. The proportion of long /aː/ responses for the middle steps 3, 4, and 5 is plotted in Fig. 5, which shows that the proportion of long /aː/ responses increases with duration step (recall that higher steps indicate longer vowel durations). Furthermore, the difference between the blue dashed line and the red solid line suggests that word lists in the bisyllabic condition (blue dashed line) induced more long/aː/responses than the monosyllabic condition (red dashed line), suggesting that vowel perception was dependent on acoustically distinct syllable rates. However, crucially, there is hardly any difference between the blue and red solid lines (i.e., between the ambiguous-as-monosyllabic and the ambiguous-as-bisyllabic conditions).

The GLMM (full model output is given in OSM Table S7) showed a significant effect of Step (*β* = 1.330, *SE* = 0.058, *z =* 2.527, *p < *.001), which indicates that higher steps (longer vowel durations) led to more long /aː/ responses in the ambiguous-as-monosyllabic condition. Crucially, the model did not find a significant difference between the ambiguous-as-monosyllabic list and the ambiguous-as-bisyllabic list (*β* = 0.039, *SE* = 0.089, *z =* 0.436, *p = *.66). That is, there was no evidence that orthographically disambiguating the ambiguous lists induced different responses on the target words. Further, a model with the monosyllabic condition set as intercept showed a significant difference between the bisyllabic condition and the monosyllabic condition (*β* = 0.583, *SE* = 0.089, *z =* 6.507, *p < *.001). Participants were thus more likely to perceive a long /aː/ when the target word was embedded in a bisyllabic list compared to a monosyllabic list, showing successful rate-dependent perception in our stimuli but only for acoustically distinct rates.

The first BF analysis (H0: *β* and *SE* of the ambiguous-as-monosyllabic vs. ambiguous-as-bisyllabic effect; H1: *β* and *SE* of the monosyllabic vs. bisyllabic effect) gave a BF of 6.2 $\times$ 10^−6^, indicating substantial evidence for the null hypothesis. The second, more conservative BF analysis (H0: *β* and *SE* of the ambiguous-as-monosyllabic vs. ambiguous-as-bisyllabic effect; H1: *β* and *SE* of the monosyllabic vs. bisyllabic conditions scaled based on Experiment 1 output) gave a BF of 0.15, showing that even with the new H_1_ prior there was substantial evidence for the null hypothesis.

### General discussion

The present study examined whether syllable rate affected vowel perception through rate-dependent perception. Experiment 1 showed that orthographic disambiguation of ambiguous word lists was successful in guiding word recognition in these word lists. In Experiment 2, we further found that the proportion of long /aː/ responses on trials with bisyllabic word lists was higher than on trials with monosyllabic word lists, showing successful rate-dependent perception with the present stimuli. However, crucially, we found that the proportion of long /aː/ responses on trials with ambiguous word lists did not differ when paired with bisyllabic orthography compared to monosyllabic orthography. Thus, we did not find evidence for listeners using orthographic information in an implicit rate-dependent perception task.

These findings from Experiment 2 contrast with previous studies that found an effect of linguistic information on rate perception using explicit rate judgements (Chen et al., 2025; Koreman, 2006; Plug et al., 2022, 2023). For example, Plug et al. (2023) found that acoustically identical sentences paired with bisyllabic orthography (cf. our ambiguous-as-bisyllabic condition) were perceived as faster compared to the same sentences paired with monosyllabic orthography (cf. our ambiguous-as-monosyllabic condition). However, recall that these effects in explicit rate judgements were already small and subtle. It thus seems that when listeners are explicitly instructed to actively compare the rates of two alternatives, linguistic information can play a small role in rate perception. However, the present study did not find evidence that this effect translates to vowel perception in an implicit task. This might suggest that during rate-dependent perception, which has been suggested to operate prior to attentional processes (Bosker et al., 2020), listeners do not use linguistic information about the intended syllable rate.

Importantly, this result does not seem to be driven by an inability of orthography to disambiguate the acoustically ambiguous word lists. That is, as Experiment 1 showed, the orthographic disambiguation was successful in guiding word recognition (e.g.., distinguishing *klom* from *kolom*) in the word lists. Yet, listeners used only the acoustic and not the intended syllable rate in the implicit task in Experiment 2. Furthermore, the effect of orthography on word recognition in Experiment 1 seemed to be less fragile compared to the effects of orthography on explicit rate judgements in previous tasks (cf. Plug et al., 2023). This difference is presumably driven by different task demands: Experiment 1 in the present study tested for *word recognition* (i.e., which word did you hear?) while Plug et al. (2023) tested for *rate judgements* (i.e., which of two sequentially presented stimuli was faster?). Note that regardless of where exactly these differences stem from, the interpretation of the results from Experiment 2 remains unchanged: Even with the large effect of orthography on word recognition observed in Experiment 1, participants relied mostly on the acoustic syllable rate in Experiment 2.

Crucially, however, based on the present study, we cannot firmly conclude that linguistic information does not affect rate-dependent perception at all. An important reason for this is that the present study cannot differentiate between whether orthography affected speech perception prelexically or post-perceptually (Cutler et al., 1998; Pattamadilok et al., 2007; Ziegler et al., 2003). That is, while the results of Experiment 1 showed that orthography affected which context word was perceived, it tested for word recognition in a forced-choice task and could therefore be driven by both prelexical and post-perceptual effects of orthography. Hence, the interpretation of the outcomes of Experiment 2 changes depending on whether orthography is assumed to operate prelexically or post-perceptually. On the one hand, if orthography affects speech perception post-perceptually, then the null result in Experiment 2 can be explained by an inability of orthography to affect prelexical rate-dependent perception (Reinisch & Sjerps, 2013) specifically because of the post-perceptual locus of orthographic effects. On the other hand, if orthography does affect perception prelexically, then the null result would more likely be driven by a more general inability of orthographic information to affect rate-dependent perception. Importantly, regardless of which of these interpretations is correct, both of them suggest that orthographic information about how many syllables a word has, as implemented in Plug et al. (2023), does not translate to an implicit rate-dependent perception task.

There are two other possible explanations for the null result. First, the effect we were targeting was a relatively small one. That is, as the results from Pilot 2 (see OSM section 1.3) and Experiment 2 indicate, the acoustically distinct rates in the present study (clear duration-matched monosyllabic and bisyllabic word lists) already resulted in a relatively small effect on vowel perception. Thus, any potential modulation by linguistic information on the ambiguous word lists would have been quite limited to begin with (i.e., it would be very unlikely to have exceeded the effect of the acoustically distinct rates).

Second, another possible explanation is that the /ɑ-aː/ duration continua in the present study were not perfectly ambiguous. More specifically, it could be that the perceptual range of the three critical middle steps was not ambiguous enough. Therefore, the effect of the orthographic disambiguation might have been effectively suppressed because the middle steps were already biased towards a mono- or bisyllabic interpretation. We would, however, argue that this is unlikely because, on those same middle steps, Experiment 2 was successful in detecting a small effect of the acoustically distinct rates. Therefore, even though we did not have perfectly ambiguous continua, the present study does show an effect of acoustic syllable rate, but not of intended syllable rate, on the same duration continua.

It is still possible, however, that different types of linguistic information do affect rate-dependent perception (Chen et al., 2025; Morrill et al., 2015; Reinisch, 2016). For instance, Reinisch (2016) found that naturally produced fast sentences with schwa deletions and assimilations were perceived as faster than linearly time-compressed sentences of the same duration but without fast-speech processes, as indexed by the proportion of long vowel responses in an implicit task. In other words, the results in Reinisch (2016) seem to suggest that listeners make use of other forms of linguistic information during rate-dependent perception.

The main difference between Reinisch (2016) and the present study that might underlie these diverging results is that Reinisch (2016) could have been targeting a more salient type of linguistic knowledge formed through long-term experience. That is, Reinisch (2016) pointed out that the observed results do not necessarily suggest that participants were comparing the intended and actual acoustic rate, as was targeted in the present study, but instead participants relied on prior knowledge that speech produced with fast-speech processes is an indication of a higher speech rate. Note, however, that a more recent study (Kahloon et al., 2023) failed to find evidence for the “reverse” of this effect: clear speech, as produced for instance when speaking to a hearing-impaired listener, is typically slower than conversational speech (i.e., contains “slow-speech processes”; Picheny et al., 1985). Because of this slow speaking rate, the perception of a /d-t/ VOT continuum is biased towards shorter /d/ when it is preceded by (slower) clear speech versus (faster) conversational speech. However, clear speech (i.e., containing “slow-speech processes”) was not found to induce more/d/responses than conversational speech artificially slowed down to match the clear speech (i.e., without “slow-speech processes”; Kahloon et al., 2023). Perhaps listeners need sufficient amounts of exposure before they can use knowledge about fast-speech/slow-speech processes implicitly and early in perception. Support for this idea can be found in Baese-Berk et al. (2014), who showed that rate effects that stem from tracking a talker’s global speech rate (i.e., the variation and rate over an extended period of time) became stronger over time. Again, this suggests that rate information that goes beyond the immediate, distal acoustic context needs time and experience before it affects rate-dependent perception. In contrast, the present study required trial-by-trial integration of the acoustic and intended rates. Thus, it is possible that throughout the experiment, there was not enough long-term exposure and it needed more attention and/or cognitive control, while rate-dependent perception has been found to operate before such processes (Bosker et al., 2017, 2020; Reinisch & Sjerps, 2013).

Turning to the question of which mechanisms (domain-general or domain-specific) drive rate-dependent perception, we did not find evidence for the involvement of domain-specific mechanisms. Our findings are thus more in line with domain-general accounts of rate-dependent perception. Note that these accounts do not imply that rate-dependent perception is driven solely by the acoustics (cf. Bosker & Reinisch, 2015, 2017; Chen et al., 2025; Maslowski et al., 2019; Morillon & Schroeder, 2015; Pitt et al., 2016; Reinisch, 2016). As Bosker et al. (2017) point out, higher-level influences can still play a role, but might only do so at a later point in time (Maslowski et al., 2020) or at the time when a decision has to be made on a target sound. It is likely that such effects could include influences from linguistic information, but the present study highlights that this might need to include pre-existing knowledge about how that linguistic information is associated with speech rate (cf. Reinisch, 2016). Future research is required, however, to formally test this possibility.

To conclude, the present study successfully established that orthography can influence word recognition (Experiment 1). However, we did not find evidence that orthographically derived linguistic information about the intended syllable rate affected vowel recognition through rate-dependent perception (Experiment 2). This outcome is more in line with domain-general than with domain-specific accounts of rate-dependent perception.

## Supplementary Information

Below is the link to the electronic supplementary material.

Supplementary file1 (DOCX 1059 KB)

## Data Availability

The stimuli, experimental data, and analysis scripts of this study are publicly available at 10.34973/kw23-aa86 under a CC-BY-4.0 license.

## Declarations

### Open practices statement

The stimuli, experimental data, and analysis scripts of this study are publicly available at 10.34973/kw23-aa86 under a CC-BY-4.0 license.
