# Supplementary material for: Is rate-dependent perception affected by linguistic information about the intended syllable rate?
Source: Psychon Bull Rev. 2025 Sep 25;32(6):3286–99. doi: 10.3758/s13423-025-02746-x (PMC12627188; doi:10.3758/s13423-025-02746-x)
Supplement: Supplementary file 1 — Supplementary file1 (DOCX 1059 KB) [file 13423_2025_2746_MOESM1_ESM.docx]

Supplementary Information

Is rate-dependent perception affected by linguistic information about the intended syllable rate?

Giulio G.A. Severijnen ^a^, Hans Rutger Bosker ^a,b^, and James M. McQueen ^a,b^

*^a^ Donders Institute for Brain, Cognition and Behaviour, Radboud University, Nijmegen, the Netherlands*

*^b^ Max Planck Institute for Psycholinguistics, PO Box 310, 6500 AH, Nijmegen, The Netherlands*

**This Supplementary Information file includes:**

1. Supplementary text
   1. Context word pilot (Pilot 1)
   2. Target word manipulations
   3. Target word pilot (Pilot 2)
   4. Testing the acoustic rates (Pilot 3)
   5. Analyses on full duration continuum
2. Supplementary tables S1-S12
3. Supplementary figures S1-S6

**1. Supplementary text**

- 1. **Context word pilot (Pilot 1)**

After stimulus manipulations, we had 8-step continua for 10 context words that ranged from a monosyllabic word on step 1 (e.g., *klom,* /klɔm/) to a bisyllabic word on step 8 (e.g., *kolom*, /ko.ˈlɔm/. Note that these 8-step continua originated from an 18-step continuum and we selected steps 1, 3-8, and 18 for the context word pilot (Pilot 1). For this pilot we recruited 24 participants (7 female, 14 male, *M_age_* = 25.23, range *=* 19-34) from the Prolific participant pool (Palan & Schitter, 2018) that did not participate in either experiment in the present study.

The aim of this pilot was to select the best steps on the continua that would serve as an ambiguous item midway between mono- and bisyllabic. The pilot was built and hosted using the Gorilla Experiment Builder ([www.gorilla.sc](http://www.gorilla.sc)). We ran a two-alternative forced choice (2AFC) experiment in which participants were presented with the context words in isolation, and instructed to respond to whether they heard the monosyllabic or bisyllabic item. The trial structure was as follows, participants first saw a fixation cross in the middle of the screen. After 500 ms, we auditorily presented a token of the context words. At sound offset, two options appeared on the screen (e.g., *klom* and *kolom*), and participants were instructed to respond with button presses ([Z] or [M] for the left and right option respectively) indicating which word they had heard. If no response was given after 3 s from sound offset, the trial was recorded as a missing data point. The next trial started 1 s after the response or after the timeout in case of a missing data point. For each context word, we presented the middle steps (steps 3 – 8) four times and the extreme steps (steps 1 and 18) once, to increase the number of observations but still provide solid anchors. This resulted in a total of 260 trials (10 context words, 4 repetitions of 6 middle steps, 1 repetition of 2 extreme steps).

For the analyses, we calculated the proportion of bisyllabic responses on each step for each context word separately (see Supplementary Figure S1). Based on these results, and auditory evaluations of the first author, we selected the step that was closest to a proportion of .50. We did this separately for each word because, as Figure S1 shows, the categorization curves differed between words. The mean proportion of bisyllabic responses, averaged across context words, was .52, showing that we managed to find items that were ambiguous between mono- and bisyllabic.

- 1. **Target word manipulations**

We obtained recordings from a female native talker of Dutch, who produced the target words in isolation. For the experiment, we eventually needed a 7-step duration continuum ranging from a short /ɑ/ (step 1) to a long /aː/ (step 7). Since in Dutch, the /ɑ-aː/ vowel contrast is cued by both spectral and durational cues (Adank et al., 2004), we also needed to selected ambiguous values for the first and second formant (F1; F2).

To obtain these continua, we first measured the mean vowel duration, mean F1, and mean F2, averaged across target words, in clear long and short vowels, as well as averaged across long and short vowels (see Supplementary Table S4). This provided information on which values signal clear short, long, and ambiguous vowels between /ɑ/ and /aː/ for this particular talker.

We then created a 7 x 7 continuum grid for each target word (see Supplementary Figure S2), in which on one axis we manipulated vowel duration, ranging from a short /ɑ/ (short duration; step 1) to a long /aː/ (long duration; step 7). On the second axis, we manipulated the vowel F2, again ranging from a short /ɑ/ (low F2; step 1) to a long /aː/ (high F2; step 7). For the duration continua, we manipulated vowel length using PSOLA in Praat (Boersma & Weenink, 2019), which ranged from 72 ms (step 1) to 203 ms (step 7) in 7 equal steps of 21.8 ms. For the F2 continua, we first set the F1 to an ambiguous value of 777 Hz (based on the average of acoustic measurements on a vs. aa items) using Burg’s LPC method in Praat. Then, using the same method, we increased the vowel F2 from 1305 Hz (step 1) to 1648 Hz (step 7) in 7 equal steps of 49 Hz. This resulted in 49 unique tokens for each target word: on 7 different vowel lengths, ranging from short to long, there were 7 tokens that varied from a low F2 to a high F2.

Based on auditory evaluations of the first author, we found that the middle step on the F2 continua did not always correspond to the perceptually most ambiguous token, possibly due to the spectral properties of the original recordings. That is, the same F2 manipulation might result in perceptually different stimuli depending on the F2 in the original recording. We thus selected a unique ambiguous middle F2-step for each target word separately (see Supplementary Table S5 for the steps and the unique F2 value), and shifted the F2 continua to the new ambiguous step being the middle step.

- 1. **Target word pilot (Pilot 2)**

The target word pilot (Pilot 2) had three main goals. First, it informed us on how the manipulated target words would be perceived. Specifically, we assessed whether the extreme steps (steps 1 and 7) would indeed be perceived as a clear short /ɑ/ or a long /aː/, and which middle steps along the duration and F2 continua would be perceived as most ambiguous. Second, this pilot provided us with the opportunity to run an experiment in which we only presented clear monosyllabic and bisyllabic word lists. This would inform us on whether these stimuli would be able to elicit a rate-dependent perception effect on the target words, indicating a perceived rate difference between the clear monosyllabic and bisyllabic word lists before introducing the ambiguous lists in the main experiment. Third, to test whether the manipulated target words were susceptible to a rate effect in the first place, we also presented word lists in which the context words were linearly expanded (to create a slow version) or compressed (to create a fast version).

In the pilot, we did not test all 49 tokens on the 7 x 7 continuum grids, but presented only a subset of the steps. Specifically, we tested only the extreme steps to provide anchor points to the participants, as well as the 9 most ambiguous steps (steps 3, 4, and 5 on the duration continuum paired with steps 3, 4, and 5 on the F2 continuum). We only tested these steps because we expected, based on auditory evaluations from the first author, that these would be closest to ambiguity.

We created the word lists by sampling the same combinations of context words as in the main experiment. The only difference between this pilot and the main experiment is that the target words appeared in list-final position in this pilot. The word lists were additionally manipulated with PSOLA in Praat (Boersma & Weenink, 2019) to create a fast version using linear compression (factor of 0.625) and a slow version using linear expansion (factor of 1.6). This resulted in six different versions (monosyllabic and bisyllabic in slow, normal, and fast rates) of each word list. Similar to the main experiment, each word list was linked to one target word, and we concatenated each step of that target word to the word list. This resulted in 396 unique stimuli (6 word lists $\times$ 3 rates $\times$ 2 syllable conditions $\times$ 11 target word steps).

The pilot (*N* = 20, 13 female, 7 male*, M_age_* = 27.80, range = 18-65; recruited from the Radboud University participant pool; none of them participated in either experiment in this study) was built and hosted on the Gorilla Experiment Builder ([www.gorilla.sc](http://www.gorilla.sc)). The trial structure was similar to that in Experiment 2. However, we did not present the trials in two separate blocks, but participants received all conditions fully interleaved in pseudo-randomized order.

For descriptive analyses, we first calculated the proportion of long /aː/ responses, across all rate conditions (slow, normal, fast) and across the syllable conditions (monosyllabic, bisyllabic), but separately for each duration and F2 step (see Supplementary Figure S3). This illustrated that the extreme steps (steps 1 and 7) were indeed perceived as a clear short /ɑ/ and a long /aː/. Further, this showed that the F2-step 3 was closest to ambiguity. We thus selected step 3 for the main experiment (see Supplementary Table S5 for mean F2 values).

Second, for further descriptive analyses, we calculated the proportion of long /aː/ responses on each step, divided by rate condition (see Supplementary Figure S4) and syllable condition (see Supplementary Figure S5), informing us on whether a rate effect would be observed with these target words (between slow, normal and fast lists) and whether we could induce a rate effect with the syllable conditions (between monosyllabic and bisyllabic lists). As Supplementary Figure S4 shows, the fast word lists (red line) resulted in more /aː/ responses than the middle (green line) and slow (blue line) word lists. On the other hand the difference between the middle and slow word lists was numerically much smaller, or even appeared to be absent on F2 steps 4 and 5. Regarding the syllable conditions, Supplementary Figure S5 shows that the bisyllabic lists did result in more /aː/ responses than the monosyllabic lists, but this effect was not consistent across all F2s. Moreover, the effect was numerically smaller compared to the rate effect between the fast and normal word lists.

To statistically test this, we ran a Generalized Linear Mixed model (GLMM) with a logistic linking function in the lmerTest package (Kuznetsova et al., 2017) in R (R Core Team, 2020). We only analyzed the data for the middle duration steps (steps 3, 4 and 5), and only for F2 step 3, as we selected this step for the main experiment. This resulted in 2421 observations. The model took the categorization of the target words as binomial dependent variable (long vowel /aː/ coded as 1; short vowel /ɑ/ coded as 0). The fixed factors were Duration Step (continuous predictor, scaled to z-scores), Rate Condition (categorical variable with three levels, intercept is middle rate), and Syllable Condition (categorical variable with two levels, deviance coded with monosyllabic coded as -0.5 and bisyllabic coded as 0.5). A model with an interaction between duration step and syllable condition or between duration step and rate condition did not improve the fit to the data. The model further included random intercepts for participants and by-item random slopes for Duration Step.

The model showed a significant effect of Duration Step (*β* = 1.284, *SE* = 0.131, *z* = 9.789, *p* < .001), indicating that higher steps (longer vowel durations) led to more long /aː/ responses. Further, the model showed a significant difference between the fast rate and the middle rate (*β* = 0.632, *SE* = 0.149, *z* = 4.215, *p* < .001), but no significant difference between the slow rate and the middle rate (*β* = -0.210, *SE* = 0.149, *z* = -1.448, *p* = .147). This shows that the fast rate word lists led to more long /aː/ responses compared to both the middle and slow rate word lists, but the middle and slow rate led to similar proportions of long /aː/ responses. Finally, and most importantly, the model showed a significant effect of Syllable Condition (*β* = 0.253, *SE* = 0.120, *z* = 2.104, *p* < .05), suggesting that the bisyllabic word lists led to more long /aː/ than the monosyllabic word lists.

Returning to the three main goals of the pilot, these results show that (1) step 3 on the F2 continuum was the F2 step that was closest to ambiguity, (2) clear monosyllabic and bisyllabic word lists induced rate-dependent perception of vowel duration, as indicated by more long /aː/ responses in the bisyllabic condition, and (3) the manipulated target words are susceptible to a rate effect, as shown by the difference between the rate conditions and syllable conditions.

**1.4 Testing the acoustic rates (Pilot 3)**

Having established that the target words were suitable for the present study, we ran one final pilot (Pilot 3) to test whether changing the position of the target word to a non-final position would increase the effect of Syllable Condition. We recruited 20 participants (8 female, 12 male, *M_age_* = 25.7, range = 18-33*;* recruited from the Prolific participants pool (Palan & Schitter, 2018); none of them participated in either experiment in the present study). This pilot tested new word lists in which the target words appeared in non-final position. Moreover, we included only target word tokens from the most ambiguous F2 step 3. These word lists were later used as the monosyllabic and bisyllabic conditions in Experiment 2.

The experiment was again a 2AFC experiment, built and hosted on the Gorilla Experiment Builder ([www.gorilla.sc](http://www.gorilla.sc)), with the same trial structure as Pilot 2. Figure S6 shows the proportion of long /aː/ responses on the middle steps (3, 4 and 5), separately for the monosyllabic and bisyllabic conditions. This shows that the bisyllabic lists seem to induce a more long /aː/ responses compared to the monosyllabic lists.

We tested this using a GLMM with a logistic linking function in the lmerTest package (Kuznetsova et al., 2017) in R (R Core Team, 2020). Similar to Pilot 2, we only analyzed the data for the middle duration steps, resulting in 4287 observations. The model took the categorization of the target words as binomial dependent variable (long vowel /aː/ coded as 1; short vowel /ɑ/ coded as 0). The fixed factors were Duration Step (continuous predictor, scaled to z-scores) and Syllable Condition (categorical variable with two levels, deviance coded with monosyllabic coded as -0.5 and bisyllabic coded as 0.5). A model with an interaction between duration step and syllable condition or between duration step and rate condition did not improve the fit to the data. The model further included by-participants random slopes for Duration Step and random intercepts for item.

The model showed a significant effect of Duration Step (*β* = 1.477, *SE* = 0.083, *z* = 17.860, *p* < .001), indicating that higher steps (longer vowel durations) led to more long /aː/ responses. Further, the model showed a significant difference of Syllable Condition (*β* = 0.454, *SE* = 0.084, *z* = 5.415, *p* < .001), suggesting that the bisyllabic word lists led to more long /aː/ than the monosyllabic word lists. Moreover, the effect was statistically more reliable than the one in Pilot 2. Therefore, we used these word lists with the target words in non-final position in the present study.

**1.5. Analyses on full duration continuum**

The GLMM (full model output is given in Supplementary table S8) showed a significant effect of Step (*β* = 3.630, *SE* = 0.054, *z* = 66.537, *p* < .001), which indicates that higher steps (longer vowel durations) led to more long /aː/ responses in the ambiguous-as-monosyllabic condition. Crucially, the model did not find a significant difference between the ambiguous-as-monosyllabic list and the ambiguous-as-bisyllabic list (*β* = -0.009, *SE* = 0.065, *z* = -0.139, p = .89). That is, there was no evidence that orthographically disambiguating the ambiguous lists induced different responses on the target words. Further, a model with the monosyllabic condition set as intercept showed a significant difference between the bisyllabic condition and the monosyllabic condition (*β* = 0.550, *SE* = 0.085, *z* = 6.432, *p* < .001). Participants were thus more likely to perceive a long /aː/ when the target word was embedded in a bisyllabic list compared to a monosyllabic list, showing successful rate-dependent perception in our stimuli but only for acoustically distinct rates.

The first BF analysis (H0: β and SE of the ambiguous-as-monosyllabic vs. ambiguous-as-bisyllabic effect; H1: β and SE of the monosyllabic vs. bisyllabic effect) gave a BF of 7.0 × 10^-7^ indicating substantial evidence for the null hypothesis. The second, more conservative BF analysis (H0: β and SE of the ambiguous-as-monosyllabic vs. ambiguous-as-bisyllabic effect; H1: β and SE of the monosyllabic vs. bisyllabic conditions scaled based on Experiment 1 output) gave a BF of 0.03, showing that even with the new H1 prior there was substantial evidence for the null hypothesis.

**1.6 Analyses on different participant samples**

Participant recruitment in Experiment 2 was divided over two different platforms with 32 participants being recruited from the Radboud University participant pool and 40 participants from Prolific (Palan & Schitter, 2018). To check whether these two samples were comparable, we analyzed the data from each sample separately.

We first computed the proportion of long /aː/ responses for the different duration steps for each condition. These are provided in Tables S9-10. While these are to some extent informative, it is difficult to conclude how variable/comparable each sample is. For that reason, we also analyzed the data from each sample by running the same GLMM as in Experiment 2. Importantly, both analyses show the same effects as in Experiment 2 (for full model output, see Tables S11-12), illustrating comparable outcomes across the two recruitment platforms in Experiment 2.

**2. Supplementary tables**

**Table S1.**

Dutch word pairs (in Dutch orthography, IPA transcriptions, and English translations) used as context words. Each word pair contains a monosyllabic and a bisyllabic word.

| Monosyllabic item (Dutch) | IPA transcription | Monosyllabic item (English) | Bisyllabic item (Dutch | IPA transcription | Bisyllabic item (English) |
| --- | --- | --- | --- | --- | --- |
| *klom* | klɔm | (I) climbed | *kolom* | ko.ˈlɔm | column |
| *fruit* | frœyt | fruit | *vooruit* | fɔ.ˈrœyt | forward |
| *trein* | trɛin | train | *terrein* | tɛ.ˈrɛin | terrain |
| *fluit* | flœyt | flute | *voluit* | fɔ.ˈlœyt | entirely |
| *breit* | brɛit | (he/she) knits | *bereid* | bə.ˈrɛit | willing (to) |
| *gleed* | ɤlet | (he/she) slid | *geleed* | ɤə.ˈlet | articulated |
| *grijs* | ɤrɛis | grey | *gereis* | ɤə.ˈrɛis | travelling |
| *groep* | ɤrup | group | *geroep* | ɤə.ˈrup | calling |
| *flop* | flɔp | failure | *volop* | fɔ.ˈlɔp | entirely |
| *klos* | klɔs | spool | *kolos* | ko.ˈlɔs | colossus |

**Table S2.**

Dutch word pairs (in Dutch orthography, IPA transcription, and English translations) used as target words. Each word pair contains one word with a long vowel and one with a short vowel.

| Long vowel (Dutch) | IPA transcription | Long vowel (English) | Short vowel (Dutch | IPA transcription | Short vowel (English) |
| --- | --- | --- | --- | --- | --- |
| *daad* | daːt | deed | *dat* | dɑt | that |
| *kaak* | kaːk | jaw | *kak* | kɑk | poop |
| *staaf* | staːf | bar | *staf* | stɑf | rod |
| *staat* | staːt | state | *stad* | stɑt | city |
| *taak* | taːk | task | *tak* | tɑk | branch |
| *zaad* | zaːt | seed | *zat* | zɑt | (he/she) sat |

**Table S3.**

Target list combinations with four context words and one target word (in Dutch orthography). For each combination, there was a monosyllabic, bisyllabic, and an ambiguous version.

| Monosyllabic word list | Bisyllabic word list | Ambiguous word list |
| --- | --- | --- |
| *klom, fruit, trein, dat/daad, fluit* | *kolom, vooruit, terrein, dat/daad, voluit* | *k?lom, v?ruit, t?rein, dat/daad, v?luit* |
| *breit, gleed, grijs, kak/kaak, groep* | *bereid, gleed, gereis, kak/kaak, geroep* | *b?reid, g?leed, g?reis, kak/kaak, g?roep* |
| *klos, fluit, flop, staf/staaf, breit* | *kolos, voluit, volop, staf/staaf, bereid* | *k?los, v?luit, v?lop, staf/staaf, b?reid* |
| *gleed, trein, breit, stad.staat, klom* | *geleed, terrein, bereid, stad/staat, kolom* | *g?leed, t?rein, b?reid, stad/staat, k?lom* |
| *fruit, groep, fluit, tak/taak, grijs* | *vooruit, geroep, voluit, tak/taak, gereis* | *v?ruit, g?roep, v?luit, tak/taak, g?reis* |
| *trein, flop, klos, zat/zaad, gleed* | *terrein, volop, kolos, zat/zaad, geleed* | *t?rein, v?lop, k?los, zat/zaad, g?leed* |

**Table S4.** Mean acoustic measures of vowel duration and F2, averaged across target words

| Cue | Short /ɑ/ | Ambiguous | Long /aː/ |
| --- | --- | --- | --- |
| *Vowel duration (ms)* | *115* | *181* | *246* |
| *F2 (Hz)* | *1354* | *1501* | *1648* |

**Table S5.** Overview of the new middle ambiguous step and the F2 values of the new continua. These were selected based on auditory evaluations made by the first author.

| Word pair | New middle step | Step 1 F2 (Hz) | Step 3 F2 (Hz) | Step 4 F2 (Hz) | Step 5 F2 (Hz) | Step 7 F2 (Hz) |
| --- | --- | --- | --- | --- | --- | --- |
| *dat – daad* | *4* | *1354* | *1452* | *1501* | *1550* | *1648* |
| *kak – kaak* | *3* | *1395* | *1493* | *1452* | *1501* | *1599* |
| *staf – staaf* | *3* | *1395* | *1493* | *1452* | *1501* | *1599* |
| *stad – staat* | *4* | *1354* | *1542* | *1501* | *1550* | *1648* |
| *tak – taak* | *1* | *1207* | *1305* | *1354* | *1403* | *1501* |
| *zat – zaad* | *4* | *1354* | *1542* | *1354* | *1550* | *1648* |

**Table S6.**

Generalized Linear Mixed model output of Experiment 1. The final model was glmer(categorization context words ~ Condition + (1 | Participant) + (1 | Item). The ambiguous-as-monosyllabic condition is set to the intercept.

| Fixed effect | *β* | *SE* | *z* | *p* |
| --- | --- | --- | --- | --- |
| (Intercept) | -0.63 | 0.33 | -1.94 | = .05 |
| Condition (ambiguous-as-bisyllabic) | 2.63 | 0.15 | 17.77 | < .001 |
| Condition (bisyllabic) | 5.09 | 0.26 | 19.52 | < .001 |
| Condition (monosyllabic) | -3.09 | 0.20 | -15.59 | < .001 |

**Table S7**

Generalized Linear Mixed model output of Experiment 2. The final model was glmer(categorization target words ~ Condition + Step + Trial Number (1 + Step + Trial Number | Participant) + (1 +Trial Number | Item). The ambiguous-as-monosyllabic condition is set to the intercept.

| Fixed effect | *β* | *SE* | *z* | *p* |
| --- | --- | --- | --- | --- |
| (Intercept) | -1.33 | 0.53 | -2.52 | < .05 |
| Condition (ambiguous-as-bisyllabic) | 0.04 | 0.09 | 0.44 | = .66 |
| Condition (bisyllabic) | 0.30 | 0.07 | 4.54 | < .001 |
| Condition (monosyllabic) | -0.28 | 0.09 | -3.11 | < .001 |
| Step | 1.61 | 0.06 | -27.96 | < .001 |
| Trial Number | 0.53 | 0.06 | 8.24 | < .001 |

**Table S8**

Generalized Linear Mixed model output of Experiment 2, analyzed on the full duration continuum. The final model was glmer(categorization target words ~ Condition + Step + Trial Number (1 + Step + Trial Number | Participant) + (1 +Trial Number | Item). The ambiguous-as-monosyllabic condition is set to the intercept.

| Fixed effect | *β* | *SE* | *z* | *p* |
| --- | --- | --- | --- | --- |
| (Intercept) | -1.19 | 0.47 | -2.52 | < .05 |
| Condition (ambiguous-as-bisyllabic) | -0.008 | 0.06 | -0.14 | = .89 |
| Condition (bisyllabic) | 0.27 | 0.06 | 4.27 | < .001 |
| Condition (monosyllabic) | -0.30 | 0.06 | -4.63 | < .001 |
| Step | 3.63 | 0.05 | 66.54 | < .001 |
| Trial Number | 0.50 | 0.02 | 21.29 | < .001 |

**Table S9**

Mean proportion of long /aː/ responses in Experiment 2, for participants that were recruited through the Radboud University participant pool. Means are given for each duration step and condition. *SD* are given in parentheses.

| Step | Monosyllabic | Ambiguous-as-monosyllabic | Ambiguous-as-bisyllabic | Bisyllabic |
| --- | --- | --- | --- | --- |
| 1 | .98 (.12) | .99 (.09) | .99 (.10) | .99 (.09) |
| 3 | .58 (.49) | .66 (.47) | .62 (.49) | .69 (.46) |
| 4 | .31 (.46) | .38 (.49) | .35 (.48) | .58 (.49) |
| 5 | .11 (.32) | .14 (.35) | .13 (.34) | .19 (.39) |
| 7 | .01 (.07) | .01 (.11) | .02 (.13) | .01 (.09) |

**Table S10**

Mean proportion of long /aː/ responses in Experiment 2, for participants that were recruited through the Prolific participant pool. Means are given for each duration step and condition. *SD* are given in parentheses.

| Step | Monosyllabic | Ambiguous-as-monosyllabic | Ambiguous-as-bisyllabic | Bisyllabic |
| --- | --- | --- | --- | --- |
| 1 | .98 (.12) | .98 (.14) | .98 (.12) | .98 (.14) |
| 3 | .52 (.50) | .56 (.49) | .55 (.49) | .63 (.48) |
| 4 | .25 (.43) | .27 (.45) | .30 (.46) | .32 (.47) |
| 5 | .09 (.30) | .11 (.31) | .13 (.34) | .11 (.32) |
| 7 | .01 (.06) | .01 (.06) | .02 (.08) | .01 (.06) |

**Table S11**

Generalized Linear Mixed model output of Experiment 2, for participants that were recruited through the Radboud University participant pool. The final model was glmer(categorization target words ~ Condition + Step + Trial Number (1 + Step + Trial Number | Participant) + (1 +Trial Number | Item). The ambiguous-as-monosyllabic condition is set to the intercept.

| Fixed effect | *β* | *SE* | *z* | *p* |
| --- | --- | --- | --- | --- |
| (Intercept) | -0.89 | 0.55 | -1.62 | = .11 |
| Condition (ambiguous-as-bisyllabic) | -0.04 | 0.13 | -0.27 | = .79 |
| Condition (bisyllabic) | 0.29 | 0.10 | 2.93 | < .01 |
| Condition (monosyllabic) | -0.31 | 0.13 | -2.37 | < .05 |
| Step | 1.57 | 0.08 | 20.79 | < .001 |
| Trial Number | 0.54 | 0.10 | 5.42 | < .001 |

**Table S12**

Generalized Linear Mixed model output of Experiment 2, for participants that were recruited through the Prolific participant pool. The final model was glmer(categorization target words ~ Condition + Step + Trial Number (1 + Step + Trial Number | Participant) + (1 +Trial Number | Item). The ambiguous-as-monosyllabic condition is set to the intercept.

| Fixed effect | *β* | *SE* | *z* | *p* |
| --- | --- | --- | --- | --- |
| (Intercept) | -1.72 | 0.61 | -2.83 | < .01 |
| Condition (ambiguous-as-bisyllabic) | 0.10 | 0.12 | 0.87 | = .39 |
| Condition (bisyllabic) | 0.32 | 0.09 | 3.46 | < .001 |
| Condition (monosyllabic) | -0.25 | 0.12 | -2.06 | < .05 |
| Step | 1.67 | 0.09 | 19.20 | < .001 |
| Trial Number | 0.53 | 0.08 | 6.74 | < .001 |

1. **Supplementary figures**

**
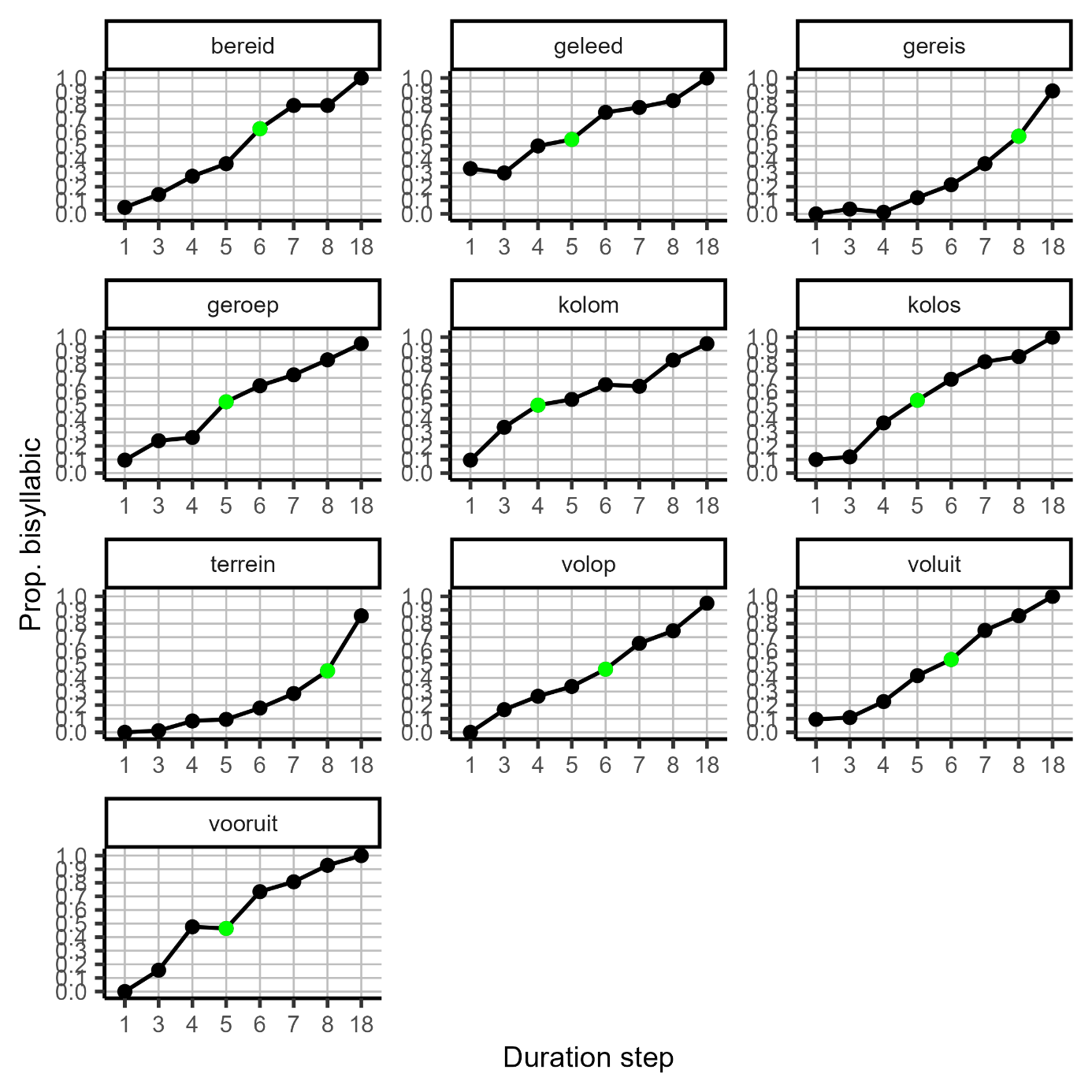
**

**Figure S1.** Categorization responses of the context word pilot (Pilot 1). Results are plotted separately for each context word. The x-axis represents the different steps on the vowel length, the y-axis represents the proportion of bisyllabic word responses. The selected step is depicted in green.


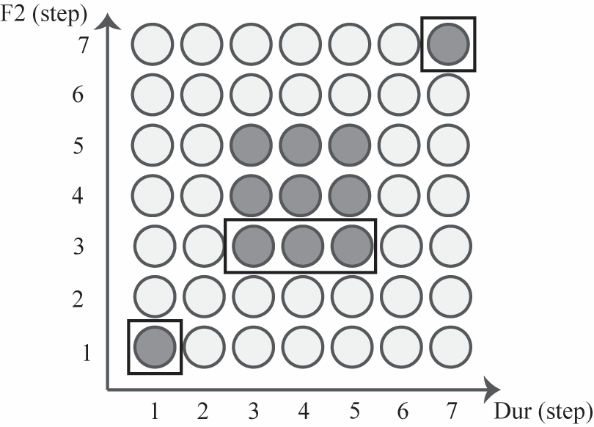


**Figure S2.** Overview of the 7 $\times$ 7 continua grid. One axis, we manipulated the vowel duration, ranging from a short /ɑ/ (step 1) to a long /aː/ (step 7). On the other axis, we manipulated the vowel F2, ranging from a low F2 (step 1) to a high F2 (step 7). The light gray circles represent all the 49 unique stimuli, the dark gray stimuli represent the stimuli that were included in the pilot. The circles surrounded by the blocks were the steps that were selected for the main experiment.


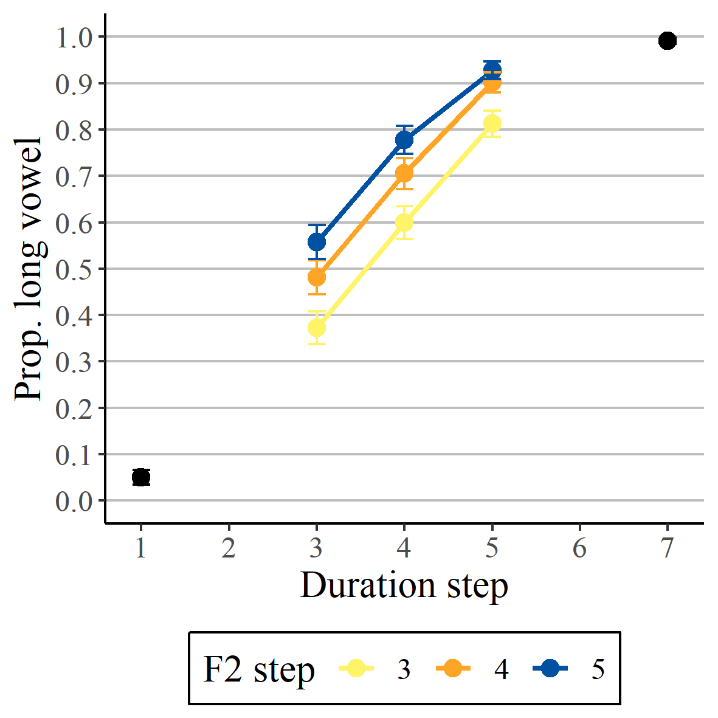


**Figure S3.** Mean categorization responses for the target word pilot (Pilot 2), averaged across rates (slow, normal, fast) and syllable condition (monosyllabic, bisyllabic). The x-axis represents the duration step, the y-axis represents the proportion of long /aː/ responses. The F2 steps are color coded. Error bars represent 95% CIs.


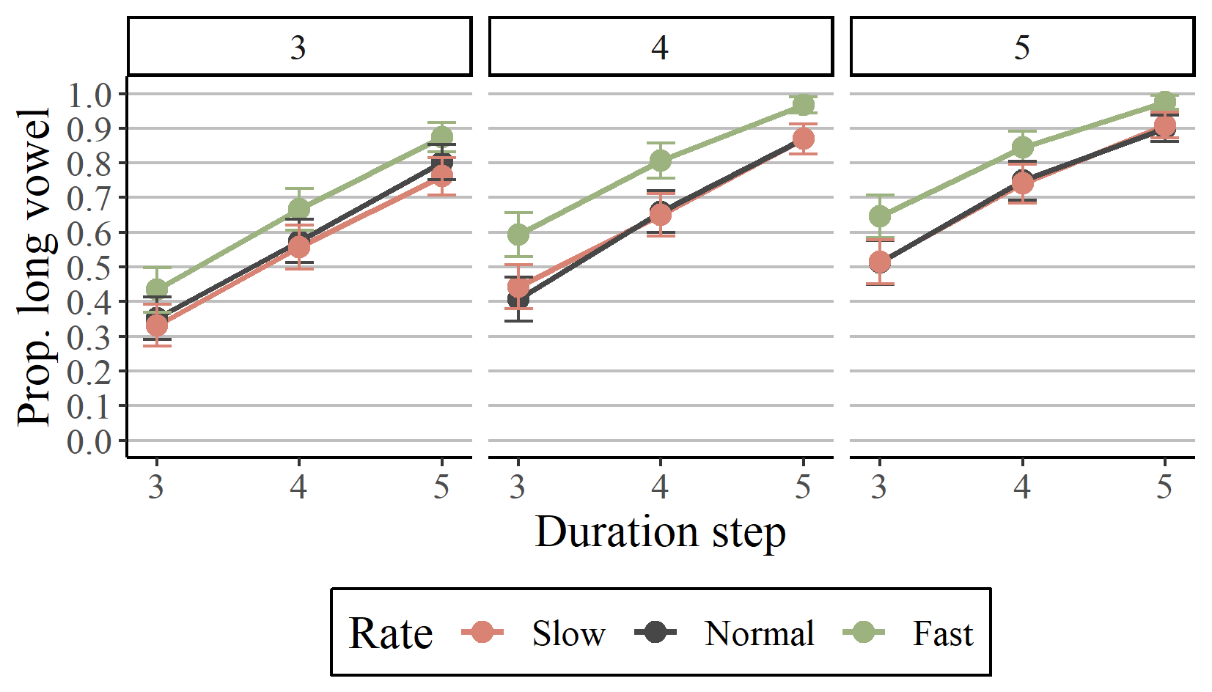


**Figure S4.** Mean categorization responses for the target word pilot (Pilot 2), split by rates (slow, normal, fast), averaged across syllable condition (monosyllabic, bisyllabic). The x-axis represents the duration step, the y-axis represents the proportion of long /aː/ responses. The rates are color coded. Each panel represents a different F2 step on the F2 continuum. Error bars represent 95% CIs.


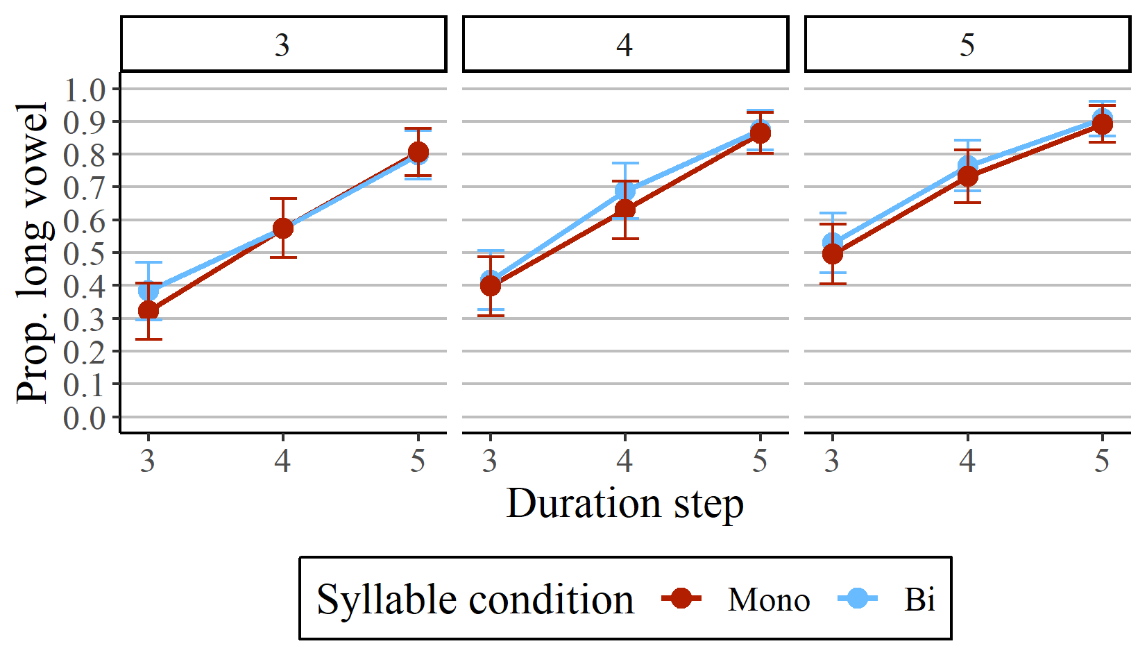


**Figure S5.** Mean categorization responses for the target word pilot (Pilot 2), in the normal rate condition, split by syllable condition (monosyllabic, bisyllabic). The x-axis represents the duration step, the y-axis represents the proportion of long /aː/ responses. The rates are color coded. Each panel represents a different F2 step on the F2 continuum. Error bars represent 95% CIs.


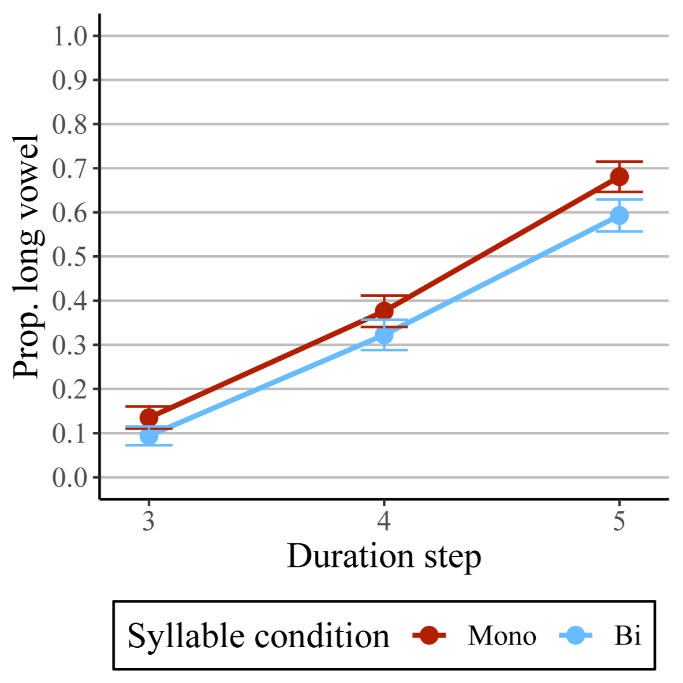


**Figure S6.** Mean categorization responses for Pilot 3 split by syllable condition (monosyllabic, bisyllabic). The x-axis represents the duration step, the y-axis represents the proportion of long /aː/ responses. The syllable conditions are color coded. Error bars represent 95% CI.
